# Evaluation of implant site preparation with piezosurgery versus conventional drills in terms of operation time, implant stability and bone density (randomized controlled clinical trial- split mouth design)

**DOI:** 10.1186/s12903-022-02613-4

**Published:** 2022-12-03

**Authors:** Hani Arakji, Essam Osman, Nayer Aboelsaad, Mohamed Shokry

**Affiliations:** 1 Oral Surgical Sciences Department, Faculty of Dentistry, Beirut Arab University, Riad El Solh, P.O. Box 11-5020, Beirut, Lebanon; 2 Oral Rehabilitation Sciences Department, Faculty of Dentistry, Beirut Arab University, Beirut, Lebanon; 3 Oral and Maxillofacial Surgery Department, Faculty of Dentistry, Alexandria University, Alexandria, Egypt

**Keywords:** Piezosurgery, Implant osteotomy, Implant stability, Bone density

## Abstract

**Background:**

The preparation of the implant bed has a major influence on the success rate and long-term survival of dental implants. Piezoelectric devices and special implant drilling inserts are now emerging to replace conventional drills showing improved bone response and healing around implants. The purpose of this study is to compare the piezoelectric inserts versus the traditional drills for implant site preparation.

**Methods:**

Twelve male patients who received a total of twenty-four dental implants have been selected to participate in this split-mouth clinical trial. Each patient received two implants; one installed after piezosurgery assisted osteotomy, while the contralateral side received the implant with the original drilling protocol. The timing of surgery, implant stability, and bone density around the installed dental implants have been evaluated during a follow-up period extended to 4 months.

**Results:**

a significant difference in terms of time of surgery (*p* < 0.005) and in implant stability at 4 months (*p* = 0.024) on the study side, while a non-statistical significance in terms of bone density was detected (*p* = 0.468).

**Conclusion:**

The piezoelectric implant site drilling protocol seemed to be a reliable and repeatable technique. Despite the limited sample size and lengthier operative time, the piezoelectric inserts enhanced bone quality and implant stability.

*Clinical trial registration* Current Controlled Trials (ClinicalTrials.gov) https://clinicaltrials.gov/ct2/show/NCT05512273; the date of registration: 23/08/2022. Retrospectively registered.

## Background

Implant site preparation is a technique-sensitive procedure. If performed a-traumatically and correctly, a promising osseointegration can be expected [1]. Conventional implant shaping drills are widely used because they are easy to handle, time-efficient, and not expensive [2]. Nevertheless, the heat that they generate might cause tissue damage, necrosis to the surrounding structures, difficulty in providing a proper three-dimensional positioning and the risk of invading and injuring important anatomical structures like the inferior alveolar nerve and the Schneiderian membrane [3–5]. Animal biomolecular and histologic studies on piezosurgery demonstrated promising signs of early bone repair after implant implantation [6]. Furthermore, other experimental investigations have suggested that ultrasonic has an impact on angiogenesis [7], the production of reparative dentin by odontoblasts, and the activation of dental pulp stem cells to develop into odontoblasts [8]. Piezosurgery appears to be more successful than drills in encouraging bone repair in periodontal and implant surgery, according to two animal pilot investigations. An ultrasonic cut causes an earlier rise in BMP-4 and TGF-b2 levels, reduces inflammation, and promotes accelerated bone remodeling [9, 10].

Piezosurgery has been introduced as an alternative to perform safer osteotomies in many surgical procedures; direct and indirect sinus elevation, bone harvesting, ridge splitting, lateralization of the inferior alveolar nerve canal, and orthognathic and neurological surgeries. It has a selective bone-cutting action without injuring soft tissues [3]. The electric charge flows into the hydroxyapatite crystals within their handpiece, leading to their deformation and creating microvibrations or ultrasonic frequency oscillations at their working tip [4]. It also increases visibility in the operative field due to the micro streaming and cavitation phenomena, which will subsequently aid in surgical precision and promote bone healing [2].

In animal studies [11], it has been suggested that implant osteotomies performed using piezosurgery result in the same amount of osseointegration. A recent systematic review comparing implant osteotomies using the conventional drilling protocol versus the piezoelectric inserts concluded that the drilling protocol, regardless of the equipment used, may not affect the implant survival rate [12]. Also, Pecker et al. reported that piezosurgery implant site osteotomies have less osteoclastic activity and more favorable bone healing, with lower RANKL levels than traditional drills [13]. Moreover, according to recent consensus, piezosurgery greatly outperforms traditional drilling methods for implant osteotomy in terms of improving the secondary stability of implants 1, 2 and 3 months after placement [14]

The rationale for conducting this study was to compare piezoelectric and conventional drilling protocols in a split mouth clinical trial to detect the impaction of piezoelectric bone cutting on the stability and bone density of dental implants. Therefore, the primary objective of this study was to evaluate the implant stability of piezosurgery osteotomy versus conventional surgical drills for implant site preparation through measuring the implant stability quotient (ISQ), and the secondary objective was to assess the operation time of the osteotomy and bone density through grey values on CBCT around the implants for both drilling methods.

The null hypothesis was stated that there is no statistically significant difference between both drilling protocols for implant site preparation.

## Materials and methods

**Fig. 1:**
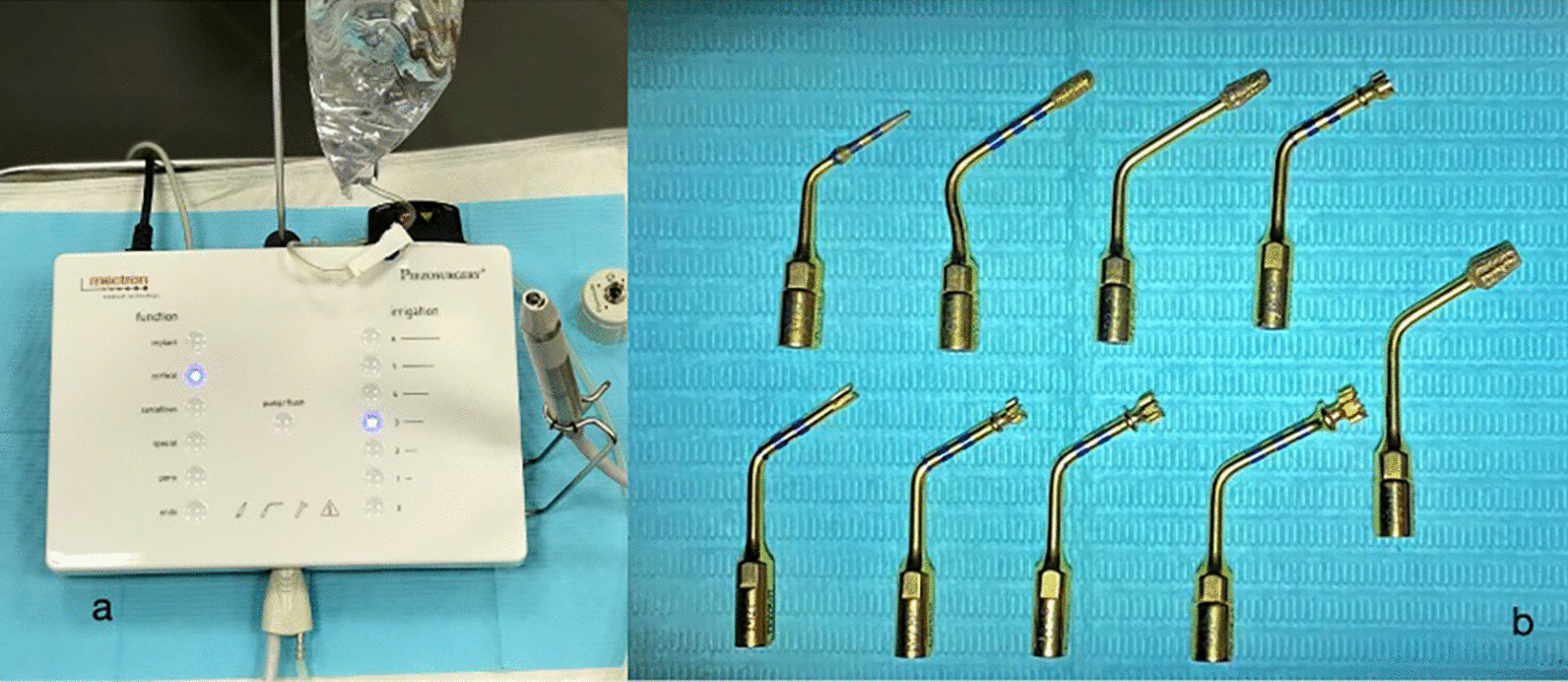
**a** Mectron piezosurgery white device, **b** implant osteotomy inserts

This study was carried out as an experimental, split-mouth design, randomized controlled clinical trial with 1:1 allocation ratio. Patients who were seeking the restoration of their extracted premolar teeth with dental implants were selected. The sample size was estimated to be twenty implants using (http://epitools.ausvet.com.au) by comparing the means and variance of osteoprotegerin (OPG) molecular system in a similar study [13] where mean for test site = 27.12 and mean for control site = 34.73, the variance was calculated to be 34.1, assuming a confidence level of 95% and a study power of 80%. To avoid attrition of the sample, four implants were added to the study. A total of twelve patients with bilateral missing single premolar teeth were included conveniently in this study to receive a total of twenty-four delayed maxillary implants. The selected samples were randomly allocated into study and control sides; each patient received two implants; one was installed after conventional drilling protocol on one side, and the other side, the implant was inserted using the piezosurgery implant specific inserts (Mectron-Italy) (Fig. 1a, b). The right and left sides of each patient were allocated randomly into a study side (S) and a control side (C). *Allocation concealment* : An assistant was responsible for giving each patient a serial number that was used for its allocation. This number was duplicated and kept in an opaque envelope with a label indicating which mouth side he/she belonged to. This envelope was kept by a trial-independent individual who was assigned the role of opening it only at the time of intervention, so that the side to which the patient was allocated was concealed from the investigator. The participants were therefore blinded to which osteotomy technique was used.

### Eligibility criteria

The inclusion criteria were: patients with bilateral missing premolars, remaining bone height of more than 10 mm, remaining bone width of 6 mm, and male patients with an age ranging from 30 to 50 years old. On the other hand, the exclusion criteria were: patients who smoke more than 10 cigarettes per day with uncontrolled systemic disease and the presence of bone pathology within the future osteotomy site.

The selected sample was collected and operated in the outpatient clinic of Oral Surgical Sciences Department, Faculty of Dentistry, Beirut Arab University-Lebanon.

All work was conducted in accordance with the Declaration of Helsinki 1975, as revised in 2000. A detailed consent was revised and signed by each patient. Before the start of the study, an ethical approval of the Beirut Arab University Institutional Review Board (IRB) was obtained: 2019-H-0068-D-P-0332.

### Pre-surgical phase

A detailed clinical examination was performed for each patient included in this study. After they were considered eligible to participate in this study, a panoramic radiograph and cone beam computed tomography (CBCT) (Carestream CS 9600-USA) were requested; the para-axial cuts were used to evaluate the height, width of the residual ridge and bone density using grey value in the osteotomy site.

One 2 g prophylactic antibiotic (two tablets of amoxicillin 875 mg with 125 mg clavulinic acid-Augmentin GSK, UK) 1 h prior to the surgical procedure was prescribed. In addition, all participants rinsed their mouths with povidone iodine for one minute (Mundipharma-Germany) half an hour before the start of the operation.

### Surgical phase

All surgeries were performed by the same oral and maxillofacial surgeon under complete aseptic conditions. The middle superior alveolar and greater palatine nerves were anesthetized using articaine hydrochloride 4% with adrenaline 1:100,000 (Septanest by Septodont–Canada) through a short needle mounted on a metallic cartridge syringe.

**Fig. 2:**
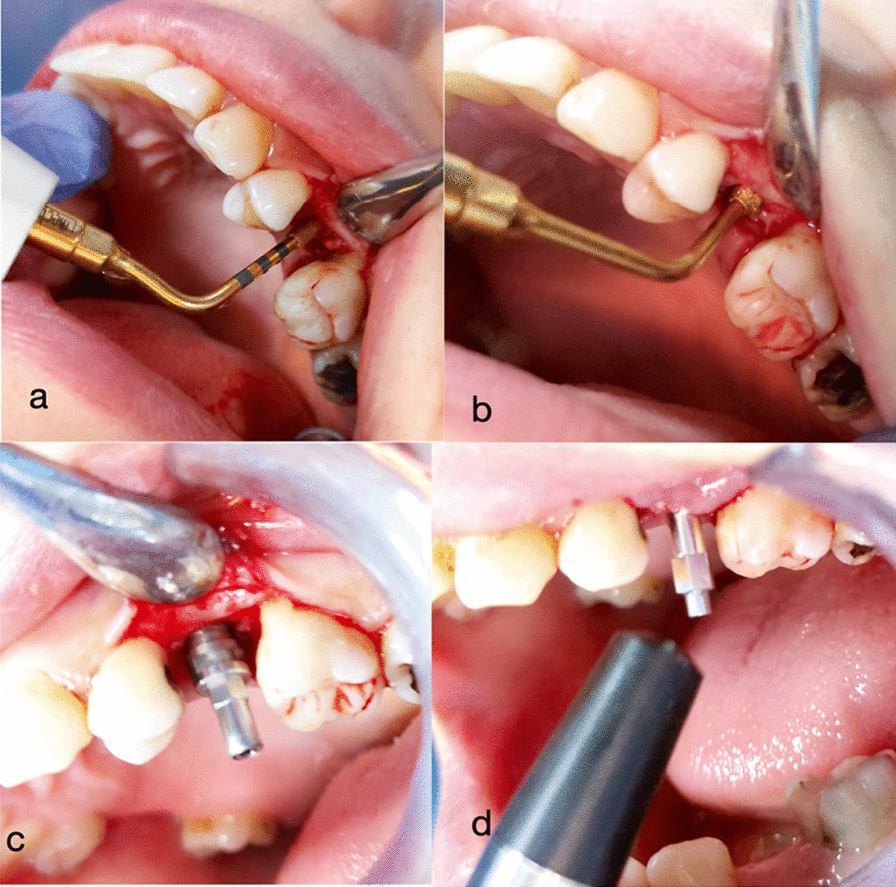
**a** OT4 insert correcting osteotomy axis, **b** P3-4 insert optimizing the concentricity of the osteotomy, **c** Bego RSX dental implant inserted, **d** Transducer probe directed towards the smartpeg to record ISQ

A full-thickness mucoperiosteal envelope flap consisted of a para-crestal incision extended sulcularly around the buccal gingival crevices of the mesial and distal neighboring teeth was elevated to access the surgical site. On the study side, sequential osteotomy drilling of the implant site for root form implants was done using the mectron piezosurgery implant drilling protocol (Mectron-Italy); first, the OT4 insert was used as a pilot drill. followed by IM3P, IM4P, P2-3, and P3-4, respectively. On the other hand, for the control side, bone osteotomy for implant placement was done following the BEGO Semados RS/RSX TrayPlus conventional drilling protocol (Fig. 2a, b).

After that, dental implants (BEGO Semados RSX, BEGO Implant Systems GmbH & Co. KG, Germany) were installed on both sides at a torque of 35–40 Ncm (Fig. 2c). The OstellTM device (Integration Diagnostics AB, Göteborg, Sweden) was used to record the primary stability insertion quotient (ISQ) and a special SmartpegTM (100,425 type 26) for BEGO Semados RSX implant system was tightened at an average of 5 Ncm torque over the implant. During pulsing, the sensor probe was pointed towards the magnet at the top of the SmartpegTM at a distance of 2–3 mm and held constant until the device beeps and showed the ISQ result (Fig. 2d). The ISQ values were taken during surgery and at 4 months post-operatively. Measurements were obtained in the buccopalatal and mesiodistal directions. The representative ISQ was determined by taking the average of the two measurements. Finally, the SmartpegTM was unscrewed, then cover screws over the installed implants were screwed properly at 10 Nc, and the flaps were repositioned and sutured using 4−0 prolene sutures.

The mobility and presence of infection of each implant were also assessed throughout the follow-up period. Also, the time of surgery was recorded in minutes from the start of flap incision until the insertion of the implant. Implant stability quotient and time of surgery were recorded.

For CBCT imaging of bone density, the KODAK 9600 3D system software (Carestream Dental, Atlanta, USA) measured grey values in numbers. Parallelism of the jawbone to the reference surface was established with the orientation beam. The settings of the CBCT tube were set to 70 kV (voltage), 107 mAs (current), and the exposure time was 12 s.

Grey values change whenever the curser moves within the volume and are automatically shown on the screen. The grey values of the bone around each implant were measured buccal to the implant in three locations of interest: apical, middle, and cervical regions of the radiological implant length, and the average of the three measurements was determined [15].

**Fig. 3:**
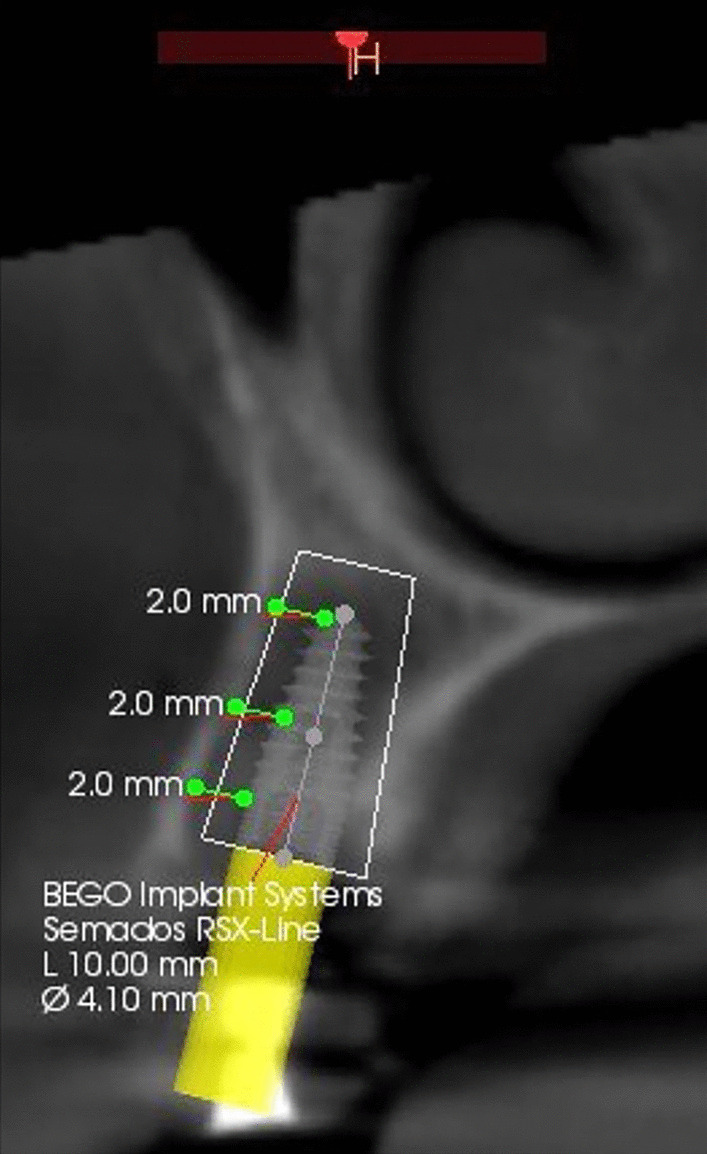
CBCT Para-axial view showing preoperative bone density measurements

A para-axial view along the center of the implant was used to assess the grey values buccally. Because the angulation of the implant couldn’t be standardized due to anatomical differences and bone morphology, the grey values and region of interest definition were recorded manually. The values were recorded at a distance of 1 mm from the implant since the titanium artifact at the bone implant interface was within 0.5 mm for all CBCT data (Fig. 3)

### Post-surgical phase

Postoperatively, 1 g (amoxicillin 825 mg with clavulinic acid 125 mg-Augmentin-GSK, UK) antibiotic twice daily was prescribed in addition to diclofenac potassium 50 mg (Cataflam-Novartis, Switzerland) three times daily for five days. Mouthwash with Chlorhexidine 0.12% (Kin gingival-Kin-Spain) was started on the second day of surgery twice daily for 10 days. Sutures were removed after 1 week.

Patients were recalled after 1 month to measure the grey value. At 4 months for the second stage surgery, the implant stability quotient was re-measured, and the bone density was re-assessed through the para-axial cuts of the CBCT. An appropriate emergence profile and gingival height healing abutment were inserted. Two weeks later, impressions needed to finalize the crown were made.

### Statistical analysis

An independent statistician reviewed the data, fed it into the computer, and analyzed it using the IBM SPSS software package version 24.0. IBM Corporation, Armonk, New York. The Kolmogorov-Smirnov test was used to verify the normality of the distribution of variables; the Student t-test was used to compare two groups for normally distributed quantitative variables. A paired *t* test was assessed for comparison between two periods for normally distributed quantitative variables. The significance of the obtained results was judged at the 5% level.

## Results

This study was carried out on twelve male patients who had a mean age of 42.12 (± 5.03) years old and sought the restoration of their missing premolar teeth with delayed dental implants. A total of twenty-four BEGO Semados RSX dental implants were inserted bilaterally.

During the regular recall visits, all the patients had uneventful healing with no signs of infection or peri-implantitis throughout the whole follow-up period.

**Fig. 4:**
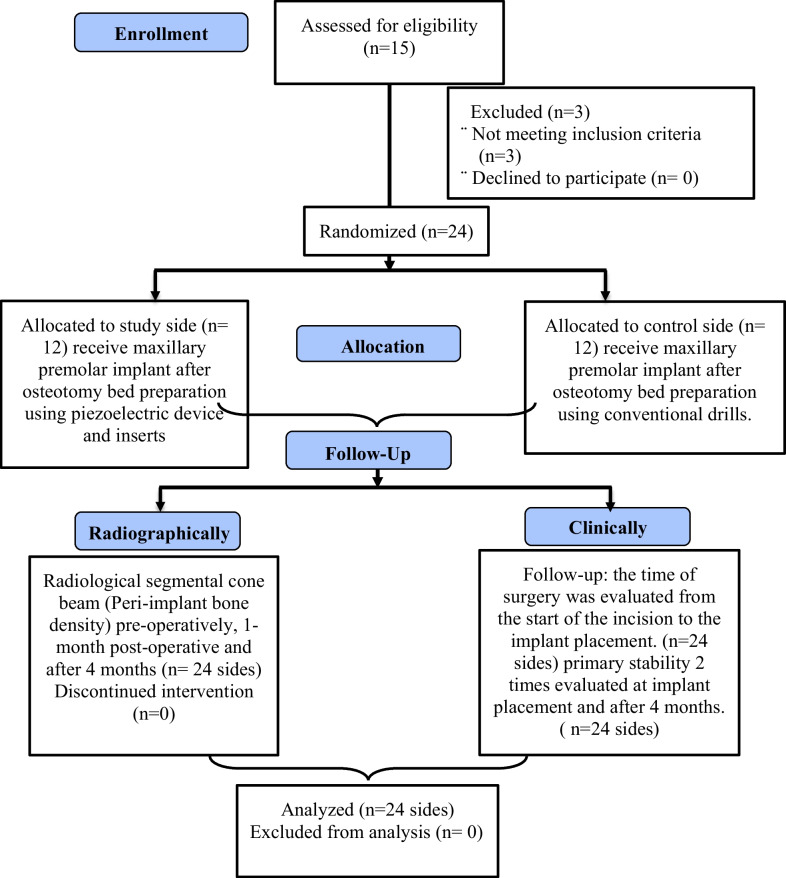
Participant flow diagram

The variables tested in this study were: operation time, implant stability quotient, and bone density around dental implants (Table 1; Fig. 4).

**Table 1:** Demographic data and sample distribution

Case number	Age	Study side	Control side
1	37	Right first premolar	Left first premolar
2	45	Left second premolar	Right first premolar
3	41	Left second premolar	Right first premolar
4	45	Right second premolar	Left first premolar
5	39	Left second premolar	Right first premolar
6	37	Left first premolar	Right first premolar
7	41	Left second premolar	Right first premolar
8	38	Left first premolar	Right first premolar
9	42	Left second premolar	Right first premolar
10	43	Right second premolar	Left first premolar
11	44	Left second premolar	Right second premolar
12	40	Right first premolar	Left first premolar

### Time of surgery

**Fig. 5:**
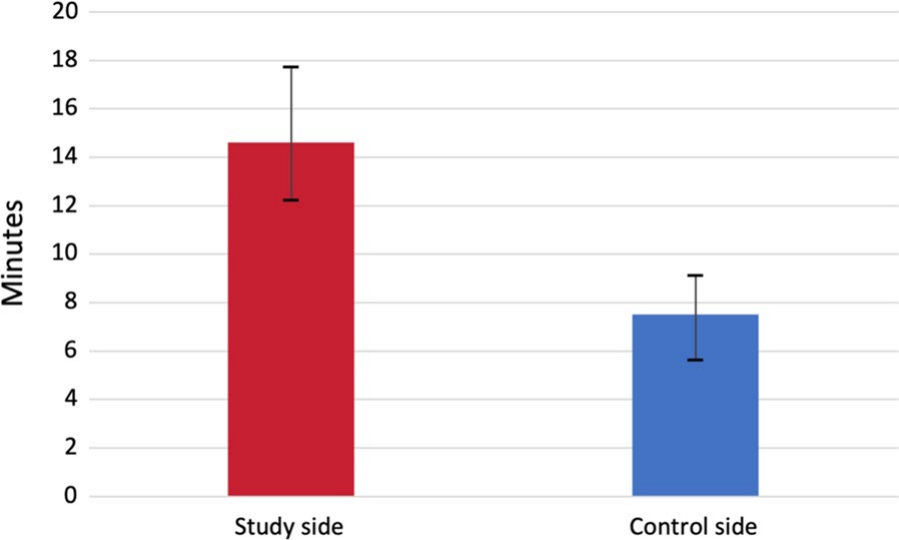
Bar chart graph comparing time in minutes

According to the assessment of the average surgical times for implant site preparation in both sides (study and control), the average time on the control side was 7.5 min (± 1.01), whereas the average time on the study side was 14.6 min (± 1.63) (Fig. 5).

When comparing the time results between both groups, a statistically significant increase in time was detected (*p* < 0.005) on the study side.

### Implant stability

Table 2 shows the comparison of implant stability quotient (ISQ) between study and control sides at different follow-up periods at the time of implant placement and after 4 months. There is no statistically significant difference in ISQ measures between the study and control sides at the time of implant placement (*p* = 0.303). The mean values of ISQ at the time of placement were higher on the study side (65.89) than on the control side (64.56). After 4 months, there was a statistically significant difference in ISQ measures between the study and control (*p* = 0.024). The study side recorded a higher mean (73.11) than the control side (70.33).

**Table 2:** Comparison between the two studied sides according to ISQ

ISQ	Mean (SD)		*p* Value of paired t test
	Study side (n = 12)	Control side (n = 12)	
At implant placement	65.89 (3.3)	64.56 (1.81)	0.303
After 4 months	73.11 (2.85)	70.33 (1.73)	0.024*
*p*_0_Value of paired t test	< 0.001*	< 0.001*	

*p*: *p* value for comparing between the two studied groups

*p*_0_: *p* value for comparing between the two periods

*: Statistically significant at *p* ≤ 0.05

### Bone density

**Fig. 6:**
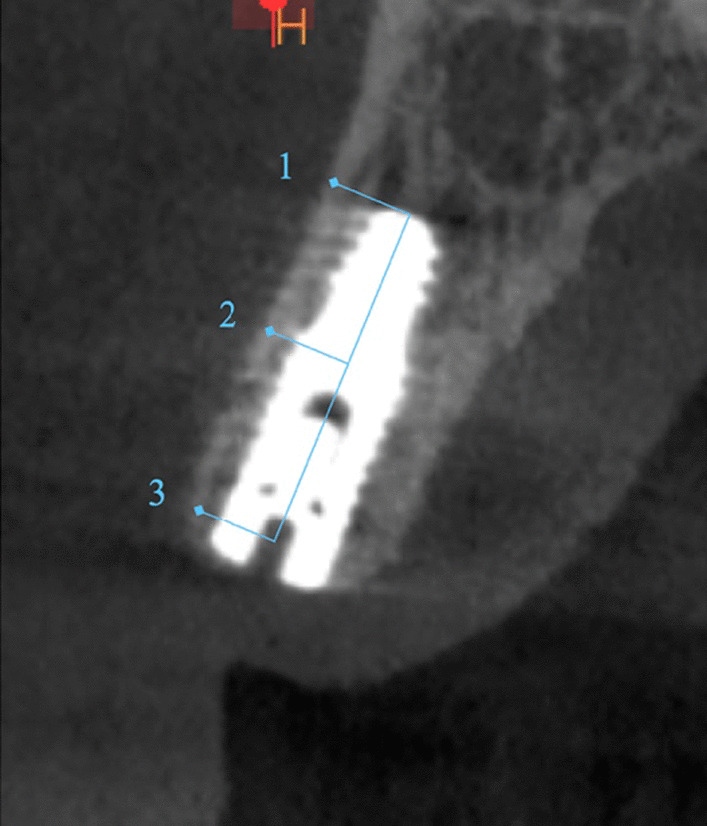
Grey value measured, apical (1), middle (2), and cervical (3). Para-axial view of the buccal measurements. The arrow indicates the region of the reference value at the lip/cheek area

**Fig. 7:**
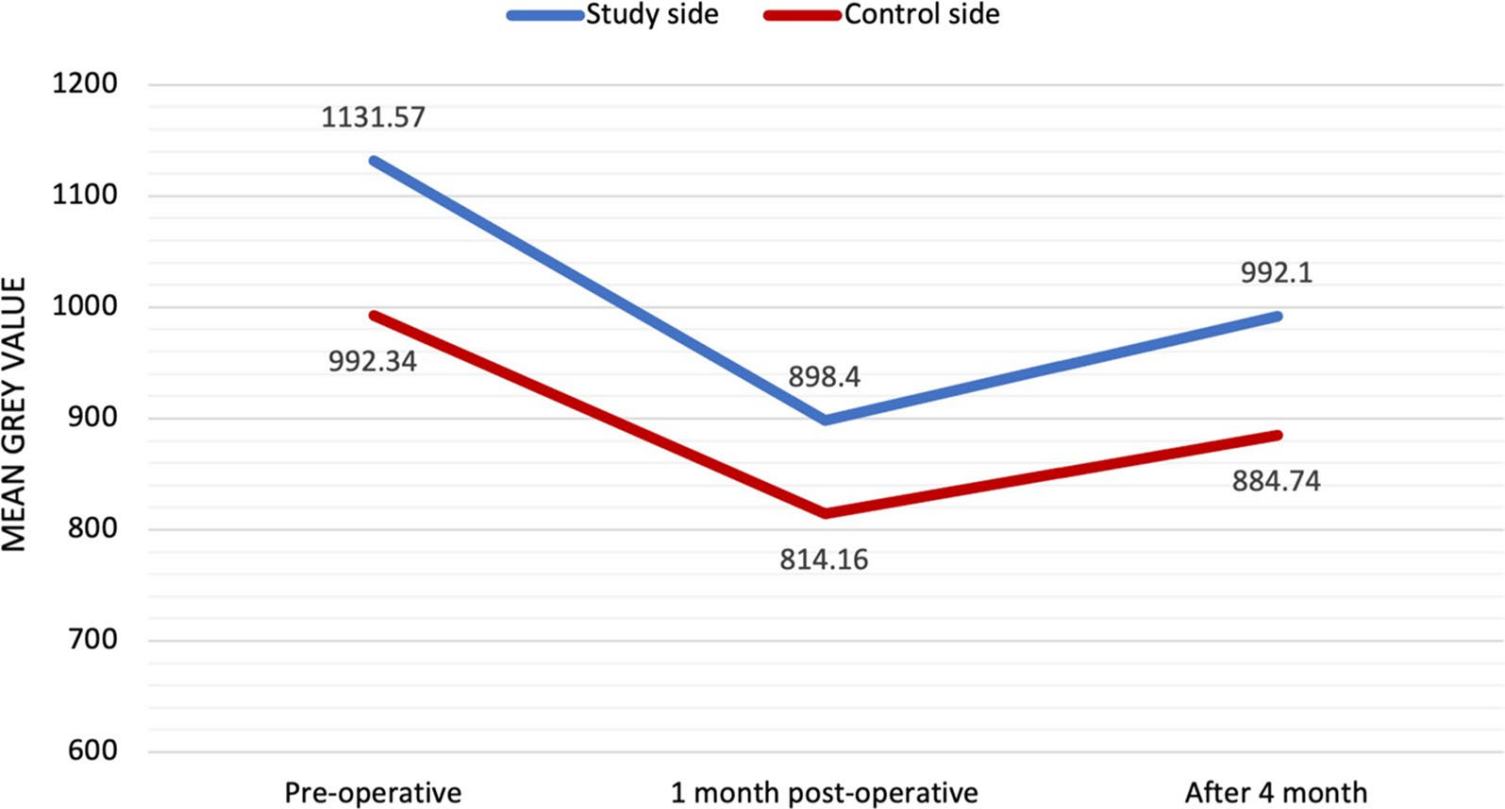
Line chart graph representing the bone density grey value at different follow up period

The mean bone density on the study side preoperatively was 1131.57, which was higher than the mean bone density of the control side (992.34) and changed over time; bone density at 1 month post-operatively was 898.40 for the piezoelectric study side and 814.16 for the conventional drilling side. At 4 months post-operatively, the mean bone density has increased from the previous follow-up interval on both sides, recording 992.10 for the study side and 884.74 for the control side. There is no statistically significant difference (*p* > 0.05) after comparing the findings between the control and study sides throughout the follow-up periods (Figs. 6 and 7)

## Discussion

Factors affecting long-term implant success and proper osseointegration are the presence of viable bone in intimate contact with the implant and the absence of implant movement when it is fully inserted. The uprise of piezoelectric devices in dentistry proved their efficacy in maintaining the vitality of the bone due to their selective cutting [16, 17]. According to certain research, piezoelectric bone surgery stimulates cell proliferation and bone synthesis, speeding up the healing process [18–20]. These benefits lead to safer crestal osteotomy, as the waving phenomena that are usually present in rotational handpieces are eliminated by the shape of the piezoelectric pivoting handpiece. Thus, it makes the initial crestal osteotomy more accurate through its cavitational phenomenon [16, 17]. Furthermore, Micro-vibrations and the cavitation action of saline solution may aid in the rapid migration of osteoprogenitor cells into a fresh wound by successfully eliminating bone fragments and tissue remains left over during osteotomy and thus promoting early healing [21].

One of numerous surgical variables that may affect the success of dental implants is the osteotomy preparation [22]. Drills that are precisely designed for each implant design are used to prepare conventional implant osteotomy. Drilling using sharp drills in the proper order under copious irrigation is crucial for preserving the vitality of the osteomatized bone, since stress caused by increased pressure and heat can hamper healing and result in implant failure [23]. Piezoelectric surgery has been proposed as a viable alternative to conventional rotational drills, which have several drawbacks [24].

Using piezoelectric inserts, we were able to compare them to conventional bone drilling procedures in terms of time of implant surgery, implant stability (ISQ), and bone density through a split mouth design and with only male patients to remove any selection bias.

Clinically, to determine the difference in surgical time, statistical analysis revealed a significant difference between the two procedures; osteotomies drilled using the piezoelectrical inserts took longer time than those performed by conventional surgical drills. These findings run parallel to those presented by Stelzle et al. and Sagheb et al. [17, 25]. A recent study stated that even if the difference was statistically significant, it may be dismissed as clinically inconsequential if surgical or biological benefits outweighed it [26]. It is still unknown if the minor difference in mean operation time between the two approaches reflects a clinical benefit for either the surgeon or the patient [26, 27].

Several methods were described to assess the primary stability of an implant. Measurements taken from the insertion torque are not repeatable. The aforementioned values were disparaged as they give an indication of the rotational force [28]. As for the percussion test, it causes patient discomfort, and the periotest lacks accuracy as it does not give repeatable results. For more accountable and reliable results as well as being more patient-friendly, the resonance frequency analysis (RFA) was used as it assesses the lateral support of the implant in bone. In fact, RFA reproducibility and simplicity make it easy to use [29].

In this study, there was a non-statistically significant difference in ISQ measures between the study and control sides at the time of implant placement. Results of this clinical trial ran in parallel to a meta-analysis that compared ISQ values between piezosurgery and conventional drilling for implant site preparation, showing a decrease in stability in the first three weeks followed by a significant increase over the healing period [12]. Stacchi et al. revealed that there is an initial slower decrease and early rise in ISQ levels [21]. Also, a systematic review and meta-analysis [31] comparing primary and secondary implant stability between piezosurgery osteotomy versus conventional drilling found a non-statistical significance between both groups but a lower decrease in implant stability ISQ when piezosurgery is used, with higher ISQ values in second readings. Nevertheless, after 4 months, a statistical difference in favor of the piezosurgery side was found. These results are in accordance with a study conducted by da Silva et al. [31], and a recent meta-analysis results with significantly higher ISQ levels in secondary implant stability [27]. These findings may indicate that although a significant difference is sometimes absent between both groups, piezosurgery osteotomy can positively affect the osseointegration of the implant due to its favorable biological results in decreasing proinflammatory cytokines activity and promoting osteoprogenitor synthesis as shown in several studies [10, 32, 33].

A preoperative examination of bone quality is required for the clinician to design a treatment plan for successful long-term implant survival. With the use of accurate bone density data, the surgeon will be able to select acceptable implant locations and determine implant design and surgical procedures. The grey value levels of CBCT were used in this study to assess bone density. Although the CT scan offers higher accuracy for bone density detection, the CBCT is office/patient-friendly with lower radiation exposure and time [34]. Soardi et al. compared CBCT and micro-CT to assess bone density after sinus lift and discovered that the CBCT grey value is comparable and predictable to the CT HU unit [35].

After interpreting the results of the current study regarding the changes in bone density around the installed dental implants, by the piezoelectric inserts versus the conventional drilling protocol, it was noted that there was a decrease in bone density grey levels after one month of implant placement on both sides. This is justified by the trauma that normally happens during osteotomy. The levels of bone density increase again to reach approximately normal preoperative values on both sides after 4 months. Although the changes in bone density levels throughout the follow-up periods showed a non-statistically significant difference among both sides, study side showed a higher grey values throughout follow up period. This is due to less heat generation on bone tissue, thus improving bone vitality; better compliance with the activity of osteoblasts; and probable preservation of soft tissues and any delicate anatomical structures next to the osteotomy [16, 17]. These findings are not in accordance with Di alberti et al. 2010 and this may be attributed to the different bone density assessment method and sample size [36].

A study while examining the correlation between ISQ and Hounsfield values of CBCT revealed a good relationship between bone density and the survival of dental implants [37]. On the other hand, Degidi et al. discovered a statistically negligible link between resonant frequency analysis results and mineralized bone–implant contact [38]. Also, a recent study conducted by Al Jamal and Al Jumaily in 2021 showed the use of CBCT to detect bone density is a reliable approach that is substantially connected with primary stability as measured by the implant stability meter [39].

The researchers encountered limitations in this study; selection of the patients meeting the eligibility criteria concerning the male gender with bilateral missed premolar. Also, due to the unavailability of Hounsfield units on CBCT, only the grey values of the CBCTs were evaluated and the presence of metal artifact that could hinder or affect the CBCT reading. In addition, it would have been useful to have a stent for repeatable para-axial cuts to measure the grey value at the same location at different time points.

## Conclusion

Considering the small sample size and increased operative time, the findings of this study showed that piezosurgery can be a safer option in maintaining the vitality of bone. Preparation of the implant bed with the aid of piezoelectric inserts showed improved implant stability after 4 months. Although the grey values did not show any statistical significance, but these values were higher at different follow up periods. The piezoelectric approach is a safe and repeatable technique, with a 100% success rate in this trial. To confirm the optimistic early findings, a lengthier follow-up investigation together with histological and histochemical studies is required. Lastly, CBCT-based monitoring of alveolar bone density can be utilized as a qualitative tool for determining changes in the bone around dental implants.

## Data Availability

materials. The datasets used and/or analysed during the current study are available from the corresponding author (Hani Arakji, haniarakji@gmail.com) upon reasonable request.
